# Clinical features and prognostic impact of asymptomatic pancreatic cancer

**DOI:** 10.1038/s41598-022-08083-6

**Published:** 2022-03-11

**Authors:** Tetsuya Takikawa, Kazuhiro Kikuta, Shin Hamada, Kiyoshi Kume, Shin Miura, Naoki Yoshida, Yu Tanaka, Ryotaro Matsumoto, Mio Ikeda, Fumiya Kataoka, Akira Sasaki, Kei Nakagawa, Michiaki Unno, Atsushi Masamune

**Affiliations:** 1 Division of Gastroenterology, Tohoku University Graduate School of Medicine, Sendai, Miyagi 980-8574 Japan; 2 Department of Surgery, Tohoku University Graduate School of Medicine, Sendai, Miyagi 980-8574 Japan

**Keywords:** Cancer, Gastroenterology

## Abstract

Pancreatic ductal adenocarcinoma (PDAC) is highly lethal, and early diagnosis is challenging. Because patients who present with symptoms generally have advanced-stage diseases, analysis of asymptomatic PDAC provides invaluable information for developing strategies for early diagnosis. Here, we reviewed 577 patients with PDAC (372 diagnosed with symptoms [symptomatic group] and 205 without symptoms [asymptomatic group]) diagnosed at our institute. Among the 205 asymptomatic PDAC patients, 109 were detected during follow-up/work-up for other diseases, 61 because of new-onset or exacerbation of diabetes mellitus, and 35 in a medical check-up. Asymptomatic PDAC is characterized by smaller tumor size, earlier disease stage, and higher resectability than those of symptomatic PDAC. In 22.7% of asymptomatic cases, indirect findings, e.g., dilatation of the main pancreatic duct, triggered PDAC detection. Although pancreatic tumors were less frequently detected, overall abnormality detection rates on imaging studies were nearly 100% in asymptomatic PDAC. Asymptomatic PDAC had a better prognosis (median survival time, 881 days) than symptomatic PDAC (342 days, *P* < 0.001). In conclusion, diagnosis of PDAC in the asymptomatic stage is associated with early diagnosis and a better prognosis. Incidental detection of abnormal findings during the follow-up/work-up for other diseases provides important opportunities for early diagnosis of asymptomatic PDAC.

## Introduction

Pancreatic ductal adenocarcinoma (PDAC) is the most common pancreatic cancer, and the number of patients with PDAC is increasing^1,2^. The American Cancer Society estimates that 60,430 patients will be diagnosed and 48,220 will die of pancreatic cancer in 2021^3^. The number of deaths due to pancreatic cancer is expected to rise to 63,000 in the United States by 2030, becoming the second leading cause of cancer-related death^4^. In Japan, according to the Vital Statistics from the Ministry of Health, Labor and Welfare, 36,356 patients died of pancreatic cancer in 2019, which was the fourth leading cause of cancer-related death^5^. PDAC is one of the most lethal malignancies, with 5-year survival rates as low as 10% and 8.5% in the United States and Japan, respectively^1,2,6^. However, it has become clear that PDAC diagnosed at an early stage has a favorable prognosis^7,8^. The Japan Pancreatic Cancer Registry reported that the 5-year survival rates for patients with stage 0 and stage IA disease, as defined by the Union for International Cancer Control (UICC), were 85.8% and 68.7%, respectively^7^. In the Surveillance, Epidemiology, and End Results registry, representative of the US population, the proportion of patients with stage IA disease has increased, and the 5-year overall survival (OS) for stage IA cases improved from 44.7% in 2004 to 83.7% in 2012^8^. However, because patients with early stage PDAC account for only a small proportion of all PDAC cases, with stage IA cases accounting for only 1.8% of all cases^8^, early diagnosis of PDAC remains challenging.

Patients with PDAC who present with symptoms generally have advanced-stage disease. The incidence of incidentally detected PDAC without symptoms has been increasing owing to advances in imaging and diagnostic modalities^9–11^. A multicenter study of early stage PDAC in Japan showed that only 25% of patients with stage 0 and I PDAC were symptomatic^12^. Although the diagnosis of PDAC among asymptomatic individuals would contribute to early diagnosis and prognosis improvement, screening for PDAC in the general population is not recommended due to the absence of useful biomarkers in the early stage, low incidence of PDAC in the general population, and physical and psychological distress associated with screening tests^13,14^. Detailed analysis of asymptomatic PDAC cases would provide invaluable information for developing strategies for the early diagnosis of PDAC. However, this information is limited. Here, we reviewed the clinical characteristics of patients with asymptomatic PDAC and compared them with those of symptomatic patients.

## Results

### Characteristics of the patients with PDAC

During the study period, 652 patients were pathologically diagnosed with PDAC at our institute. We excluded patients with intraductal papillary mucinous neoplasm (IPMN) with high-grade dysplasia (n = 29), PDAC derived from IPMN (n = 21), recurrent PDAC (n = 17), incurable malignancy of other organs (n = 5), and those who received treatment for PDAC before pathological diagnosis (n = 3). Finally, 577 patients with PDAC were enrolled, of whom 372 (64.5%) were diagnosed with symptoms (symptomatic group) and 205 (35.5%) were diagnosed without symptoms (asymptomatic group).

Of the 205 asymptomatic patients, PDAC was detected during the follow-up/work-up of diseases other than PDAC in 109 patients (34 malignant diseases, 39 benign diseases, and 36 other pancreatic diseases), upon new-onset (n = 16) or exacerbation (n = 45) of diabetes mellitus (DM) in 61 patients, and in a medical check-up in 35 patients. Other pancreatic diseases included IPMN (n = 19), pancreatic cyst (n = 8), chronic pancreatitis (n = 4), acute pancreatitis (n = 2), autoimmune pancreatitis (n = 2), and pancreatic neuroendocrine neoplasm (n = 1).

The characteristics of the enrolled patients are shown in Table 1. The disease stage was stage 0 or I for 120 (20.8%) patients and stage II or higher in 457 (79.2%) patients. Among the 112 patients with stage I PDAC, 47 (42.0%) had stage IA PDAC. Two hundred and twenty-eight (39.5%) patients underwent surgical resection, of which 21 patients received surgery only, 9 received neoadjuvant treatment, 84 received adjuvant treatment, and 114 received neoadjuvant and adjuvant treatments. DM and tobacco use were the predominant risk factors observed in approximately half of patients. The median survival time (MST) was 472 days. The 1-, 3-, and 5-year OS rates were 59.2%, 24.7%, and 14.9%, respectively.

**Table 1:** Characteristics of patients with PDAC.

Variables	Patients with PDAC (n = 577)
Age, mean (SD) (years)	68.6 (9.5)
Sex, men, n (%)	303 (52.5)
BMI, median (IQR) (kg/m^2^)	22.2 (19.9–24.4)
Weight loss, yes, n (%)^a^	216 (48.4)
Tumor size, median (IQR) (mm)	31 (24–42)
**Diagnostic opportunity, n (%)**	
Symptomatic	372 (64.5)
Asymptomatic	205 (35.5)
Follow-up/work-up for other diseases	109/205 (53.2)
New-onset or exacerbation of DM	61/205 (29.8)
Medical check-up	35/205 (17.1)
**Tumor location, n (%)**	
Head	280 (48.5)
Body	190 (32.9)
Tail	105 (18.2)
Multiple	2 (0.3)
**Clinical stage, n (%)**	
0	8 (1.4)
I	112 (19.4)
II	82 (14.2)
III	135 (23.4)
IV	240 (41.6)
**Treatment, n (%)**	
Surgery	228 (39.5)
Surgery only	21 (3.6)
Surgery with neoadjuvant treatment	9 (1.6)
Surgery with adjuvant treatment	84 (14.6)
Surgery with neoadjuvant and adjuvant treatments	114 (19.8)
Chemotherapy or chemoradiotherapy	279 (48.4)
Best supportive care	66 (11.4)
Others	4 (0.7)
**Risk factors, n (%)**	
DM	294 (51.0)
Obesity (> BMI 30 kg/m^2^)	21 (3.6)
Tobacco use	292 (50.6)
Heavy alcohol consumption (> 40 g ethanol/day)^b^	69 (12.3)
Family history of pancreatic cancer^c^	68 (13.0)
IPMN/Pancreatic cyst	85 (14.7)
Chronic pancreatitis	9 (1.6)

*PDAC* pancreatic ductal adenocarcinoma, *SD* standard deviation, *BMI* body mass index, *IQR* interquartile range, *DM* diabetes mellitus, *IPMN* intraductal papillary mucinous neoplasm. ^a^Data from 446 patients. ^b^Data from 561 patients. ^c^Data from 522 patients.

### Comparison of the clinical characteristics and prognosis between symptomatic and asymptomatic patients

We compared the clinical characteristics and prognosis between the symptomatic and asymptomatic groups. The asymptomatic group was characterized by older age, less weight loss, and more frequent tumor location in the distal pancreas when compared with the symptomatic group (Table 2). Moreover, the asymptomatic group had a smaller tumor size, earlier disease stage, and higher resectability rates. Twenty-two percent (45/205) of asymptomatic cases were stage 0 or IA, whereas only 2.7% (10/372) of symptomatic cases were at these stages. Regarding the risk factors for PDAC, the asymptomatic group had a higher incidence of DM and IPMN or pancreatic cysts than the symptomatic group.

**Table 2:** Comparison of the clinical characteristics between symptomatic and asymptomatic patients.

Variables	Symptomatic group (n = 372)	Asymptomatic group (n = 205)	*P* value
Age, mean (SD) (years)	67.5 (9.9)	70.7 (8.5)	< 0.001
Sex, men, n (%)	185 (49.7)	118 (57.6)	0.07
BMI, median (IQR) (kg/m^2^)	22.2 (19.6–24.2)	22.1 (20.1–25.1)	0.15
Weight loss, yes, n (%)^a^	159 (59.3)	57 (32.0)	< 0.001
Tumor size, median (IQR) (mm)	35 (27–45)	25 (19–32)	< 0.001
**Tumor location, n (%)**			
Head	203 (54.6)	77 (37.6)	< 0.001
Body	99 (26.6)	91 (44.4)	
Tail	69 (18.5)	36 (17.6)	
Multiple	1 (0.3)	1 (0.5)	
**Clinical stage, n (%)**			
0	2 (0.5)	6 (2.9)	< 0.001
I	33 (8.9)	79 (38.5)	
(IA)	8 (2.2)	39 (19.0)	
(IB)	25 (6.7)	40 (19.5)	
II	44 (11.8)	38 (18.5)	
III	100 (26.9)	35 (17.1)	
IV	193 (51.9)	47 (22.9)	
**Treatment, n (%)**			
Surgery	96 (25.8)	132 (64.4)	< 0.001
Surgery only	4 (1.1)	17 (8.3)	
Surgery with neoadjuvant treatment	5 (1.3)	4 (2.0)	
Surgery with adjuvant treatment	34 (9.1)	50 (24.4)	
Surgery with neoadjuvant and adjuvant treatments	53 (14.2)	61 (29.8)	
Chemotherapy or chemoradiotherapy	219 (58.9)	60 (29.3)	
Best supportive care	54 (14.5)	12 (5.9)	
Others	3 (0.8)	1 (0.5)	
**Risk factors, n (%)**			
DM	172 (46.2)	122 (59.5)	0.002
Obesity (> BMI 30 kg/m^2^)	12 (3.2)	9 (4.4)	0.47
Tobacco use	187 (50.3)	105 (51.2)	0.83
Heavy alcohol consumption (> 40 g ethanol/day)^b^	47 (13.1)	22 (10.9)	0.47
Family history of pancreatic cancer^c^	47 (14.2)	21 (10.9)	0.28
IPMN/Pancreatic cyst	30 (8.1)	55 (26.8)	< 0.001
Chronic pancreatitis	3 (0.8)	6 (2.9)	0.075

*SD* standard deviation, *BMI* body mass index, *IQR* interquartile range, *DM* diabetes mellitus, *IPMN* intraductal papillary mucinous neoplasm.

^a^Data from 268 symptomatic and 178 asymptomatic patients.

^b^Data from 360 symptomatic and 201 asymptomatic patients.

^c^Data from 330 symptomatic and in 192 asymptomatic patients.

**Figure 1:**
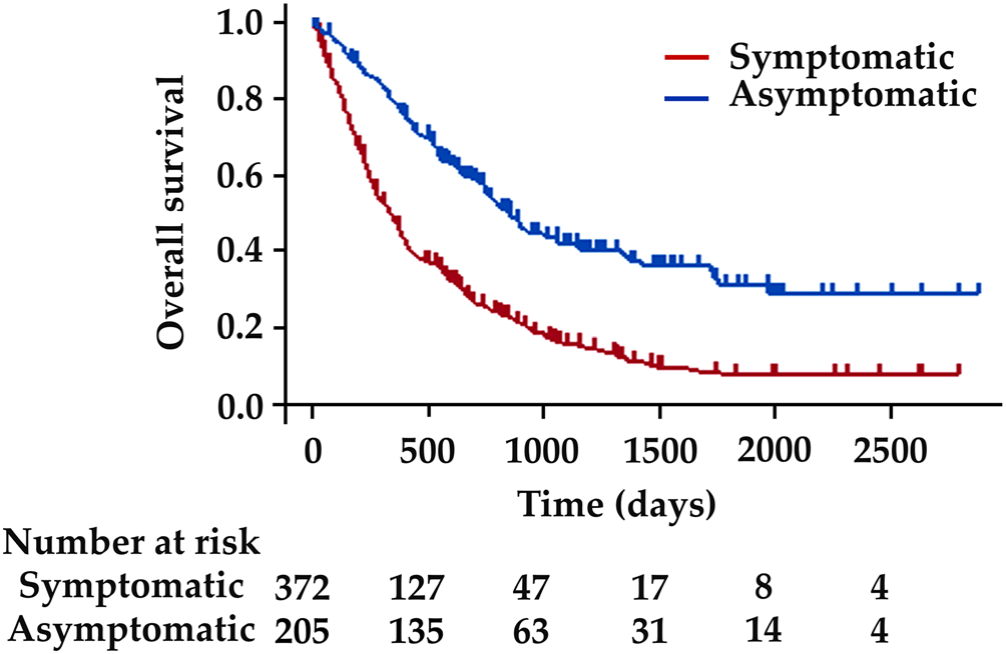
Kaplan–Meier survival curves of the patients with pancreatic ductal adenocarcinoma (PDAC) with or without symptoms. The overall survival of asymptomatic patients was better than that of symptomatic patients (*P* < 0.001).

Figure 1 shows the Kaplan–Meier estimates of the OS of patients with PDAC with or without symptoms. The 1-, 3-, and 5-year OS rates were 47.7%, 15.3%, and 6.6% in the symptomatic group and 79.8%, 41.7%, and 30.6% in the asymptomatic group, respectively. OS was longer in asymptomatic patients than in symptomatic patients; MST was 881 days in the asymptomatic group and 342 days in the symptomatic group (*P* < 0.001).

We further analyzed the factors which are associated with OS in patients with PDAC. In a univariate analysis, age, BMI, presence of weight loss, tumor size, clinical stage, treatment, neoadjuvant and adjuvant treatments, and symptoms at diagnosis were significantly associated with OS (Table 3). Among them, clinical stage, treatment, neoadjuvant treatment, and symptoms at diagnosis were extracted as significant factors associated with OS in a multivariate analysis. These results suggested that asymptomatic PDACs had better prognosis compared to symptomatic PDACs.

**Table 3:** Univariate and multivariate analyses of the factors which are associated with OS.

	Univariate analysis		Multivariate analysis	
	HR (95% CI)	*P* value	HR (95% CI)	*P* value
**Age (years)**				
< 65	1		1	
≥ 65	1.32 (1.07–1.64)	0.011	1.25 (0.96–1.62)	0.10
**Sex**				
Female	1			
Male	0.92 (0.76–1.32)	0.40		
**BMI (kg/m**^**2**^**)**				
< 18.5	1		1	
≥ 18.5, < 25	0.68 (0.50–0.92)	0.013	0.85 (0.59–1.23)	0.39
≥ 25	0.63 (0.44–0.89)	0.009	1.04 (0.68–1.60)	0.85
Presence of weight loss	1.39 (1.11–1.74)	0.004	1.08 (0.85–1.39)	0.52
**Tumor location**				
Head	1			
Body/ tail	0.98 (0.81–1.19)	0.86		
**Tumor size (mm)**				
≤ 20	1		1	
> 20	2.37 (1.77–3.17)	< 0.001	1.09 (0.73–1.63)	0.66
**Clinical stage**				
0–IA	1		1	
IB–IV	4.88 (3.00–7.94)	< 0.001	3.26 (1.66–6.38)	< 0.001
**Treatment**				
Surgery	1			
Chemotherapy or CRT	6.59 (5.12–8.49)	< 0.001	2.96 (1.53–5.73)	0.001
Best supportive care	24.7 (17.36–35.23)	< 0.001	10.88 (5.01–23.64)	< 0.001
**Neoadjuvant treatment**				
No	1		1	
Yes	0.25 (0.19–0.34)	< 0.001	0.62 (0.40–0.95)	0.027
**Adjuvant treatment**				
No	1		1	
Yes	0.18 (0.14–0.23)	< 0.001	0.65 (0.35–1.24)	0.19
**Symptoms at diagnosis**				
Symptomatic	1		1	
Asymptomatic	0.41 (0.33–0.51)	< 0.001	0.72 (0.55–0.95)	0.020

*BMI* body mass index, *CRT* chemoradiotherapy, *DM* diabetes mellitus, *HR* Hazard ratio, *OS* overall survival.

### Diagnostic opportunities and initial modalities indicating abnormalities in asymptomatic PDAC

We examined the modalities that initially indicated abnormalities leading to the diagnosis of asymptomatic PDAC. Of the 109 asymptomatic patients whose PDAC was detected during follow-up/work-up for other diseases, abnormalities were initially detected on computed tomography (CT) in 45 (41.3%) patients and on ultrasonography (US) in 27 (24.8%) (Table 4). Of the 61 patients with new-onset or exacerbation of DM, abnormalities were initially detected on CT in 30 (49.2%) patients and on US in 20 (32.8%). Of the 35 patients whose PDAC was detected in a medical check-up, abnormalities were initially detected on US in 24 (68.6%). Overall, CT was the most common modality, initially detecting abnormalities in 76/205 (37.1%) asymptomatic PDAC patients, followed by US in 71/205 (34.6%) and blood tests in 33/205 (16.1%).

**Table 4:** Modalities that initially indicated abnormalities in asymptomatic PDAC.

Initial modalities	Number (%)
**Follow-up/work-up for other diseases**	**109**
CT	45 (41.3)
US	27 (24.8)
MRI	10 (9.2)
PET/CT	8 (7.3)
EUS	3 (2.8)
Blood tests	16 (14.7)
Elevated tumor markers	12
Elevated liver enzymes	3
Elevated pancreatic enzymes	1
**New-onset or exacerbation of DM**	**61**
CT	30 (49.2)
US	20 (32.8)
MRI	2 (3.3)
Blood tests	9 (14.8)
Elevated tumor markers	9
**Medical check-up**	**35**
US	24 (68.6)
CT	1 (2.9)
MRI	1 (2.9)
PET/CT	1 (2.9)
Blood tests	8 (22.9)
Elevated tumor markers	4
Elevated liver enzymes	3
Elevated pancreatic enzymes	1
**All cases**	**205**
CT	76 (37.1)
US	71 (34.6)
MRI	13 (6.3)
PET/CT	9 (4.4)
EUS	3 (1.5)
Blood tests	33 (16.1)
Elevated tumor markers	25
Elevated liver enzymes	6
Elevated pancreatic enzymes	2

*CT* computed tomography, *US* ultrasonography, *MRI* magnetic resonance imaging, *PET* positron emission tomography, *EUS* endoscopic ultrasonography.

We then compared the initial imaging findings that triggered further examinations between the symptomatic and asymptomatic groups. In cases where US initially detected abnormalities, indirect findings in the absence of pancreatic tumor detection led to further examinations more frequently in asymptomatic cases (20/71; 28.2%) than in symptomatic cases (11/125; 8.8%) (*P* < 0.001) (Table 5). Similarly, in the case of CT, indirect findings in the absence of pancreatic tumor detection led to further examinations more frequently in asymptomatic cases (11/76; 14.5%) than in symptomatic cases (4/236; 1.7%) (*P* < 0.001). Indirect findings more frequently led to further examinations on magnetic resonance imaging (MRI) (8/13, 61.5%) than on other imaging modalities (31/159; 19.5%) (*P* = 0.001). Overall, indirect findings led to further examinations more frequently in asymptomatic patients than in symptomatic patients (22.7% vs. 4.8%) (*P* < 0.001).

**Table 5:** Initial imaging modalities and major findings that led to further examinations.

Initial imaging modalities and major findings	Symptomatic group (n = 372)	Asymptomatic group (n = 172)	*P* value
**US, n**	**125**	**71**	
Pancreatic tumors, n (%)	114 (91.2)	51 (71.8)	< 0.001
Indirect findings without tumor detection, n (%)	11 (8.8)	20 (28.2)	
**CT, n**	**236**	**76**	
Pancreatic tumors, n (%)	232 (98.3)	65 (85.5)	< 0.001
Indirect findings without tumor detection, n (%)	4 (1.7)	11 (14.5)	
**MRI, n**	**5**	**13**	
Pancreatic tumors, n (%)	2 (40.0)	5 (38.5)	1.00
Indirect findings without tumor detection, n (%)	3 (60.0)	8 (61.5)	
**PET, n**	**5**	**9**	
Pancreatic tumors with FDG accumulation, n (%)	5 (100)	9 (100)	N/A
**EUS, n**	**1**	**3**	
Pancreatic tumors, n (%)	1 (100)	3 (100)	N/A
Indirect findings without tumor detection, n (%)	0 (0)	0 (0)	
**All imaging modalities, n**	**372**	**172**	
Pancreatic tumors, n (%)	354 (95.2)	133 (77.3)	< 0.001
Indirect findings without tumor detection, n (%)	18 (4.8)	39 (22.7)	

*CT* computed tomography, *US* ultrasonography, *MRI* magnetic resonance imaging, *PET* positron emission tomography, *FDG* fluorodeoxyglucose, *EUS* endoscopic ultrasonography, *N/A* not available.

### Comparison of imaging and blood test results between symptomatic and asymptomatic patients

Table 6 shows the comparison of imaging and blood test results between the symptomatic and asymptomatic groups. All imaging modalities showed lower detection rates for pancreatic tumors in the asymptomatic group than in the symptomatic group. However, the overall detection rates of abnormal imaging findings were not different. The overall detection rates of imaging abnormalities, including pancreatic tumors, and indirect findings on US, CT, MRI, and endoscopic ultrasonography (EUS) in the symptomatic group were 90.9%, 99.7%, 99.7%, and 100%, respectively, and in the asymptomatic group were 87.2%, 100%, 99.5%, and 100%, respectively. Pancreatic tumors were not detected by EUS in 5 symptomatic and 10 asymptomatic PDAC cases. Among them, 7 cases were diagnosed by brushing cytology, serial pancreatic juice aspiration cytological examination, or pancreatic juice cytology during endoscopic retrograde pancreatography. The remaining 8 cases underwent pancreatic surgery suspected of PDAC and diagnosis of PDAC was made by postoperative pathological examination.

**Table 6:** Comparison of imaging and laboratory test results between symptomatic and asymptomatic patients.

	Symptomatic group (n = 372)	Asymptomatic group (n = 205)	*P* value
**US evaluable patients, n**	**232**	**149**	
Detection of abnormal findings, n (%)	211 (90.9)	130 (87.2)	0.25
Pancreatic tumors, n (%)	195 (84.1)	92 (61.7)	< 0.001
Indirect findings, n (%)	154 (66.4)	112 (75.2)	0.07
MPD cut-off, n (%)	115 (49.6)	69 (46.3)	0.53
MPD dilatation, n (%)	133 (57.3)	97 (65.1)	0.13
Pancreatic cysts, n (%)	45 (19.4)	37 (24.8)	0.21
**CT evaluable patients, n**	**365**	**200**	
Detection of abnormal findings, n (%)	364 (99.7)	200 (100)	0.46
Pancreatic tumors, n (%)	356 (97.5)	178 (89.0)	< 0.001
Indirect findings, n (%)	304 (83.3)	173 (87.0)	0.31
MPD cut-off, n (%)	263 (72.1)	155 (77.5)	0.16
MPD dilatation, n (%)	254 (69.6)	156 (78.0)	0.032
Pancreatic cysts, n (%)	142 (38.9)	57 (28.5)	0.013
**MRI evaluable patients, n**	**319**	**194**	
Detection of abnormal findings, n (%)	318 (99.7)	193 (99.5)	0.72
Pancreatic tumors, n (%)	299 (93.7)	149 (76.8)	< 0.001
Indirect findings, n (%)	280 (87.8)	179 (92.3)	0.11
MPD cut-off, n (%)	240 (75.2)	155 (79.9)	0.22
MPD dilatation, n (%)	232 (72.7)	157 (80.9)	0.035
Pancreatic cysts, n (%)	158 (49.5)	93 (47.9)	0.73
**EUS evaluable patients, n**	**344**	**203**	
Detection of abnormal findings, n (%)	344 (100)	203 (100)	N/A
Pancreatic tumors, n (%)	339 (98.5)	193 (95.1)	0.016
Indirect findings, n (%)	280 (81.4)	173 (85.2)	0.25
MPD cut-off, n (%)	240 (69.8)	152 (74.9)	0.20
MPD dilatation, n (%)	236 (68.6)	156 (76.8)	0.039
Pancreatic cysts, n (%)	142 (41.3)	68 (33.5)	0.07
**Laboratory test evaluable patients, n**	**372**	**205**	
Elevated tumor markers, n (%)	320 (86.0)	137 (66.8)	< 0.001
CEA, n (%)	154 (41.4)	56 (27.3)	< 0.001
CA19-9, n (%)	290 (78.0)	123 (60.0)	< 0.001
Abnormalities of pancreatic enzymes, n (%)	224 (60.2)	105 (51.2)	0.037
Elevated amylase, n (%)	49 (13.2)	34 (16.6)	0.26
Decreased amylase, n (%)	68 (18.3)	24 (11.7)	0.039
Elevated lipase, n (%)	155 (41.7)	80 (39.0)	0.54
Decreased lipase, n (%)	7 (1.9)	4 (2.0)	0.95

*US* ultrasonography, *CT* computed tomography, *MRI* magnetic resonance imaging, *EUS* endoscopic ultrasonography, *CEA* carcinoembryonic antigen, *CA19-9* carbohydrate antigen 19-9, *N/A* not available.

Regarding blood test results, elevated levels of tumor markers and abnormal pancreatic enzyme levels were more frequently observed in the symptomatic group than in the asymptomatic group. However, the positive rate for tumor markers in the asymptomatic group was not low; 137/205 (66.8%) asymptomatic patients were positive for either carcinoembryonic antigen or carbohydrate antigen 19-9 (CA19-9).

## Discussion

The major findings of this study are as follows. First, asymptomatic PDAC patients had smaller tumors, earlier disease stage, higher resectability rates, and better prognoses than those of patients with PDAC presenting with symptoms. Second, the most frequent diagnostic opportunity for asymptomatic patients was follow-up/work-up for other diseases, followed by new-onset or exacerbation of DM. Third, indirect findings such as cut-off/dilatation of main pancreatic duct (MPD) without pancreatic tumor detection led to further examinations in approximately one-quarter of patients with asymptomatic PDAC, whereas imaging revealed abnormalities in the majority of cases. This study has some limitations, including its retrospective, single-center, observational nature. Our hospital is a high-volume center for pancreatic diseases, and most patients included in this study were referred from other hospitals; therefore, there might be a selection bias from the referring physicians. Despite these limitations, our study showed that the detection of PDAC at the asymptomatic stage has a significant effect on early diagnosis and improved prognosis.

Previous studies have compared the clinical characteristics and outcomes between patients presenting with and without symptoms. Mizuno et al.^10^ analyzed 379 symptomatic patients with PDAC and 161 asymptomatic patients. They reported that asymptomatic patients had a smaller tumor size (31 vs. 38 mm), earlier disease stage, higher resectability (40 vs. 19%), and longer MST (20.2 vs. 10.2 months) than those of symptomatic patients. Takeda et al.^11^ analyzed 406 symptomatic patients with PDAC and 163 asymptomatic patients and showed that asymptomatic patients had earlier disease stage, higher resectability (64% vs. 36%), and longer MST (16 vs. 10 months) than those of asymptomatic patients. Our study is in agreement with these previous studies. In addition, we clarified the imaging findings including indirect ones in asymptomatic PDAC patients, which were not analyzed in detail in previous studies^10,11^.

In this study, PDAC was incidentally detected in 73 patients during follow-up/work-up for non-pancreatic diseases. Due to advances in imaging modalities, the prevalence of incidentally detected pancreatic lesions, so-called pancreatic incidentalomas, has been increasing during examinations for non-pancreatic lesions^15,16^. Previous studies have shown that asymptomatic PDAC was most frequently detected during follow-up/work-up for other diseases^10,11,15^. Pancreatic incidentalomas can be morphologically classified into cystic lesions, solid lesions, and MPD dilatation^16^. The prevalence of cystic pancreatic incidentalomas detected using multi-detector CT or MRI was reported to be around 2.5%^17,18^ and 9.3% when using high-resolution 3-T MRI^19^. Compared to cystic lesions, information on their prevalence is limited; however, solid pancreatic incidentalomas may be less frequently detected. Among the 333 patients who underwent evaluation for suitability for kidney donation, pancreatic masses were incidentally detected on CT in two (0.6%) subjects^20^. Among the 2868 subjects undergoing fluorodeoxyglucose (FDG) positron emission tomography (PET) scans, pancreatic lesions were detected in 14 (0.5%) subjects^21^. Solid pancreatic incidentalomas have a high malignant potential, and PDAC accounts for 31–34% of solid pancreatic incidentalomas^9,15^. When comparing between asymptomatic and symptomatic cases, cases detected with pancreatic incidentalomas had smaller tumors, earlier disease stage, higher resectability rates, and better prognoses than those detected based on symptoms^9,22^.

Incidental detection during the follow-up/work-up for other diseases is the most frequent opportunity for asymptomatic PDAC cases in this and other studies^10,11^. This point should be more widely recognized for promoting early diagnosis of PDAC. Careful attention should be paid to the pancreas when performing abdominal imaging for diseases of other organs, which may increase the number of PDAC detections. If suspicious lesions are detected, consultation to gastroenterologists should be clearly recommended in the radiology reports and the compliances to such recommendations should be followed systematically. For application of this finding to daily practice widely, it is important to clarify the frequency of PDAC detection among the subjects who undergoes abdominal imaging studies, but such information is very limited. Because most PDAC patients enrolled in this study were referred from a variety of outside hospitals, it was difficult to accurately present the total numbers of subjects undergoing these tests and that of PDAC cases diagnosed in the respective hospitals. In Japan, the estimated number of total patients who underwent CT was approximately 30 million per year, and about 6.9 million (23%) tests involved upper abdominal scans^23^. If we apply the proportion of the cases incidentally detected (18.9%) in this study to the annual incidence of PDAC (42,359 cases) in Japan in 2018^5^, about 8000 cases were detected during follow-up/work-up for other diseases. Based on this assumption, 863 abdominal CT examinations are required to detect one PDAC case. Because this number is relatively high, stratification based on risk factors for PDAC as well as application of artificial intelligence^24^ would be useful for effective screening of PDAC among subjects undergoing imaging studies.

In asymptomatic patients with PDAC, CT initially detected abnormalities in 37.1%, US in 34.6%, and blood test results in 16.1%, which in total accounted for 87.8% of all cases. These figures are similar to those of previous studies from Japan^11,25^, but different from those reported in Western countries, where asymptomatic cases have been found mainly using CT^9,26^. Bruzoni et al.^26^ reported that 89% of pancreatic incidentalomas were detected using CT. This discrepancy is presumably due to differences in the medical environment between Japan and Western countries. In Japan, US is widely available among family doctors, and patients can easily visit hospitals and clinics because of the national health insurance system. Hanada et al.^27^ reported the usefulness for the early diagnosis of PDAC of a social diagnostic system, in which family doctors actively performed US in patients with risk factors for PDAC. Regarding blood tests, although CA19-9 is not considered to be useful in the diagnosis of early stage PDAC^28^, 60% of asymptomatic patients with PDAC had elevated CA19-9 levels. Recently, Fahrmann et al.^29^ reported that CA19-9 could serve as an anchor marker for the early detection of PDAC. In the cohorts of the Prostate, Lung, Colorectal and Ovarian Cancer Screening Trial, CA19-9 levels exponentially increased starting at 2 years before diagnosis with sensitivities reaching 60% at 99% specificity within 6 months before diagnosis for all PDAC cases and 50% at 99% specificity for early PDAC. CA19-9 might be routinely evaluated in patients at high risk of PDAC.

Because population-based PDAC screening of average-risk subjects is not recommended^13,14^, screening for PDAC focuses on high-risk individuals^14,30^. New-onset or exacerbation of DM, which is a risk factor for PDAC, has been attracting attention as an important diagnostic clue^31,32^. Indeed, new-onset or exacerbation of DM served as a diagnostic clue in approximately 30% of asymptomatic PDAC cases in this study. Chari et al.^31^ reported that approximately 1% of new-onset DM patients aged > 50 years were diagnosed with PDAC within 3 years. In order to detect PDAC in a large cohort of patients with DM, development of efficient screening systems is urgently needed. Sharma et al.^33^ proposed a scoring system composed of changes in body weight, changes in blood glucose, and age at onset of DM in patients with new-onset DM. This scoring system can diagnose PDAC with 80% sensitivity and specificity.

Our study showed that the diagnosis of PDAC in the asymptomatic stage could contribute to improved prognosis due to its earlier stage and higher resectability rate. However, even if detected at an asymptomatic stage, 40% of cases were stage III or IV, and the 5-year OS was 30.6%, which is far below the average 5-year OS for all cancer types (64.1%) in Japan^6^. These figures agree with the notion that once PDAC is detectable using imaging, it is already at an advanced, possibly disseminated stage, and will rapidly progress^34^. Therefore, detecting PDAC in the asymptomatic stage is not sufficient to ensure long-term survival, and PDAC should be diagnosed at a stage when tumors are still not detectable using imaging or when they are not clinically evident. It has been increasingly recognized that indirect imaging findings, especially cut-off/dilatation of MPD in the absence of tumor detection, are important for the detection of early stage PDAC^35–37^. In a multicenter study of early stage PDAC in Japan, MPD dilatation could be detected in approximately three-quarters of the Stage 0 cases (76.5% cases on US, 72.0% on CT, and 73.9% on MRI), whereas pancreatic tumors could only be detected in approximately 10% of cases (8.8% cases on US, 10.0% on CT, and 10.9% on MRI)^12^. In addition to MPD abnormalities, recent studies have shown that focal parenchymal atrophy of the pancreas on CT serves as an important imaging sign for the early diagnosis of PDAC^38,39^. Of note, Miura et al.^39^ reported that focal parenchymal atrophy might be the earliest sign of PDAC, which appears before MPD abnormalities. Novel biomarkers, such as non-coding RNAs, exosomes, and circulating DNA, have been reported to improve the accuracy of diagnosis^40–42^.

In conclusion, we clarified the clinical features and prognosis of asymptomatic PDAC, which would be important information for the detection of such cases in daily practice. Incidental detection of abnormal findings during the follow-up/work-up for other diseases provides important opportunities for early diagnosis of asymptomatic PDAC, and this point should be more widely recognized. Further development of biomarkers, technologies, and application of artificial intelligence that allow the systematic detection of early PDAC with high sensitivity and specificity are warranted to overcome this intractable disease.

## Methods

### Subjects

We retrospectively analyzed consecutive patients who had been pathologically diagnosed with PDAC at Tohoku University Hospital between January 2013 and December 2019. We excluded patients with IPMN with high-grade dysplasia or PDAC derived from IPMN which showed a histologic transition between IPMN and PDAC^43^, those with recurrent PDAC, those who received treatment for PDAC before pathological diagnosis, and those with non-curable malignancies of other organs. Because histological features of PDAC concomitant with IPMN, but not PDAC derived from IPMN, were similar to those of ordinary PDAC^43^, we included PDAC concomitant with IPMN, in which PDAC developed at a site in the pancreas different from that of the IPMN according to the imaging and/or histologic findings^43^. The enrolled patients were classified into two groups according to the diagnostic opportunities for PDAC: subjective symptoms such as pain and jaundice as chief complaints leading to the diagnosis of PDAC (“symptomatic group”) and asymptomatic detection during follow-up/work-up for other diseases, new-onset or exacerbation of DM, or medical check-up (“asymptomatic group”). If weight loss was a chief complaint, the patient was classified to symptomatic group. If weight loss was revealed by detailed interview after asymptomatic detection, the patient was classified to asymptomatic group.

We analyzed the following factors: (a) clinical characteristics including age, sex, body mass index (BMI), weight loss, tumor location and size, clinical stage, treatment, and risk factors associated with PDAC; (b) details of diagnostic opportunities and initial examinations that led to diagnosis; (c) laboratory tests and imaging findings on abdominal US, CT, MRI/magnetic resonance cholangiopancreatography (MRCP), and EUS; and (d) long-term prognosis. Clinical, imaging, and pathological information of the patients were obtained from the medical records.

### Definition

Tumor diameters were defined as the largest diameter measured on CT or EUS at the time of initial diagnosis of PDAC. Clinical stages were classified according to the American Joint Committee on Cancer 8th edition^44^. We defined stage 0 or IA as early stage PDAC. Regarding imaging findings, indirect findings included cut-off or dilatation of the MPD and pancreatic cyst. We defined the MPD cut-off as an interruption or abrupt narrowing of the MPD^35,45^. MPD dilation was defined as a maximal MPD diameter ≥ 2 mm^15,35^. We defined OS as the time from the date of admission for PDAC to the date of the last follow-up or death.

### Follow-up

We usually followed-up patients diagnosed with PDAC using CT every 2 or 3 months and measured tumor marker levels every month. MRI/MRCP, EUS, and ^18^F-FDG PET/CT were performed as necessary.

### Statistical analysis

Continuous variables were presented as mean (standard deviation [SD]) or median (interquartile range [IQR]), and categorical variables were expressed as numbers (percentages). For comparison between two groups, Student’s t-test or Wilcoxon rank sum test was used for continuous variables, and the chi-square test or Fisher’s exact test was used for categorical variables. We used Kaplan–Meier survival analysis to compare OS. Differences in survival were evaluated using the log-rank test. We performed a multivariate analysis using the Cox proportional hazards model for the factors that were significantly associated with OS in a univariate analysis.

We used JMP Pro 15 (SAS Institute Inc., Cary, NC, USA) for statistical analysis, and a two-sided P-value < 0.05 was considered statistically significant.

### Ethical approval and consent to participate

The study was conducted according to the guidelines of the Declaration of Helsinki, and approved by the Ethics Committee of Tohoku University Graduate School of Medicine (protocol code; 2019-1-919; 2019-1-920). Patient consent was waived due to the retrospective nature of this study by the Ethics Committee of Tohoku University Graduate School of Medicine.

## References

[1] Singhi AD, Koay EJ, Chari ST, Maitra A (2019). Early detection of pancreatic cancer: Opportunities and challenges. Gastroenterology.

[2] Mizrahi JD, Surana R, Valle JW, Shroff RT (2020). Pancreatic cancer. Lancet.

[3] Siegel RL, Miller KD, Fuchs HE, Jemal A (2021). Cancer statistics, 2021. CA Cancer J. Clin..

[4] Rahib L (2014). Projecting cancer incidence and deaths to 2030: The unexpected burden of thyroid, liver, and pancreas cancers in the United States. Cancer Res..

[5] Cancer Statistics. Cancer Information Service, National Cancer Center, Japan (Vital Statistics of Japan, Ministry of Health, Labour and Welfare). https://ganjoho.jp/reg_stat/statistics/data/dl/index.html#mortality (Accessed 25 May 2021).

[6] Monitoring of Cancer Incidence in Japan—Survival 2009–2011 Report (Center for Cancer Control and Information Services, National Cancer Center, 2020). https://ganjoho.jp/reg_stat/statistics/data/dl/index.html#survival (Accessed 25 May 2021).

[7] Egawa S (2012). Japan pancreatic cancer registry; 30th year anniversary: Japan Pancreas Society. Pancreas.

[8] Blackford AL, Canto MI, Klein AP, Hruban RH, Goggins M (2020). Recent trends in the incidence and survival of stage 1A pancreatic cancer: A surveillance, epidemiology, and end results analysis. J. Natl. Cancer Inst..

[9] Lahat G (2009). Pancreatic incidentalomas: High rate of potentially malignant tumors. J. Am. Coll. Surg..

[10] Mizuno S (2013). Diabetes is a useful diagnostic clue to improve the prognosis of pancreatic cancer. Pancreatology.

[11] Takeda Y (2017). Asymptomatic pancreatic cancer: Does incidental detection impact long-term outcomes?. J. Gastrointest. Surg..

[12] Kanno A (2018). Multicenter study of early pancreatic cancer in Japan. Pancreatology.

[13] Owens DK (2019). Screening for pancreatic cancer: US preventive services task force reaffirmation recommendation statement. JAMA.

[14] Aslanian HR, Lee JH, Canto MI (2020). AGA clinical practice update on pancreas cancer screening in high-risk individuals: Expert review. Gastroenterology.

[15] Santo E, Bar-Yishay I (2017). Pancreatic solid incidentalomas. Endosc. Ultrasound..

[16] Del Chiaro M, Torphy RJ (2021). Pancreatic incidentalomas: Investigation and management. J. Intern. Med..

[17] Laffan TA (2008). Prevalence of unsuspected pancreatic cysts on MDCT. AJR Am. J. Roentgenol..

[18] de Jong K (2010). High prevalence of pancreatic cysts detected by screening magnetic resonance imaging examinations. Clin. Gastroenterol. Hepatol..

[19] de Oliveira PB, Puchnick A, Szejnfeld J, Goldman SM (2015). Prevalence of incidental pancreatic cysts on 3 tesla magnetic resonance. PLoS ONE.

[20] Strang AM (2007). Computerized tomographic angiography for renal donor evaluation leads to a higher exclusion rate. J. Urol..

[21] Pitts A, Nissen NN, Waxman A, Yu R (2013). Unsuspected fluorodeoxyglucose positron emission tomography (FDG-PET)-positive pancreatic lesions: Prevalence and significance. Pancreas.

[22] Winter JM (2006). Periampullary and pancreatic incidentaloma: A single institution's experience with an increasingly common diagnosis. Ann. Surg..

[23] Tsushima Y (2010). Radiation exposure from CT examinations in Japan. BMC Med Imaging..

[24] Mendoza Ladd A, Diehl DL (2021). Artificial intelligence for early detection of pancreatic adenocarcinoma: The future is promising. World J. Gastroenterol..

[25] Ikemoto J (2021). Clinical analysis of early-stage pancreatic cancer and proposal for a new diagnostic algorithm: A multicenter observational study. Diagnostics (Basel)..

[26] Bruzoni M, Johnston E, Sasson AR (2008). Pancreatic incidentalomas: Clinical and pathologic spectrum. Am. J. Surg..

[27] Hanada K (2015). Effective screening for early diagnosis of pancreatic cancer. Best Pract. Res. Clin. Gastroenterol..

[28] Steinberg WM (1986). Comparison of the sensitivity and specificity of the CA19-9 and carcinoembryonic antigen assays in detecting cancer of the pancreas. Gastroenterology.

[29] Fahrmann JF (2021). Lead-time trajectory of CA19-9 as an anchor marker for pancreatic cancer early detection. Gastroenterology.

[30] Canto MI (2012). Frequent detection of pancreatic lesions in asymptomatic high-risk individuals. Gastroenterology.

[31] Chari ST (2005). Probability of pancreatic cancer following diabetes: A population-based study. Gastroenterology.

[32] Takikawa T (2020). New-onset or exacerbation of diabetes mellitus is a clue to the early diagnosis of pancreatic cancer. Tohoku J. Exp. Med..

[33] Sharma A (2018). Model to determine risk of pancreatic cancer in patients with new-onset diabetes. Gastroenterology.

[34] Yu J, Blackford AL, Dal Molin M, Wolfgang CL, Goggins M (2015). Time to progression of pancreatic ductal adenocarcinoma from low-to-high tumour stages. Gut.

[35] Yoon SH (2011). Small (≤ 20 mm) pancreatic adenocarcinomas: Analysis of enhancement patterns and secondary signs with multiphasic multidetector CT. Radiology.

[36] Elbanna KY, Jang HJ, Kim TK (2020). Imaging diagnosis and staging of pancreatic ductal adenocarcinoma: A comprehensive review. Insights Imaging.

[37] Miura S (2020). Focal parenchymal atrophy and fat replacement are clues for early diagnosis of pancreatic cancer with abnormalities of the main pancreatic duct. Tohoku J. Exp. Med..

[38] Nakahodo J (2020). Focal parenchymal atrophy of pancreas: An important sign of underlying high-grade pancreatic intraepithelial neoplasia without invasive carcinoma, i.e., carcinoma in situ. Pancreatology.

[39] Miura S (2021). Focal parenchymal atrophy of the pancreas is frequently observed on pre-diagnostic computed tomography in patients with pancreatic cancer: A case–control study. Diagnostics (Basel)..

[40] Singh N (2020). Clinical significance of promoter methylation status of tumor suppressor genes in circulating DNA of pancreatic cancer patients. J. Cancer Res. Clin. Oncol..

[41] Ariston Gabriel AN (2020). The involvement of exosomes in the diagnosis and treatment of pancreatic cancer. Mol. Cancer..

[42] Dragomir MP, Kopetz S, Ajani JA, Calin GA (2020). Non-coding RNAs in GI cancers: From cancer hallmarks to clinical utility. Gut.

[43] Yamaguchi K (2011). Pancreatic ductal adenocarcinoma derived from IPMN and pancreatic ductal adenocarcinoma concomitant with IPMN. Pancreas.

[44] Amin MB (2017). AJCC Cancer Staging Manual.

[45] Campbell CH, Triggs JR, Miller FH, Komanduri S (2021). Endoscopic ultrasound for evaluation of pancreatic duct "cutoff" identified on magnetic resonance imaging improves the diagnostic yield of occult malignancy. Pancreas.

